# Prediction of Motor Recovery Using Diffusion Tensor Imaging and Regional Cerebral Blood Flow in Postoperative Brain Tumors

**DOI:** 10.7759/cureus.65099

**Published:** 2024-07-22

**Authors:** Chie Matsuura, Yuki Sakaeyama, Mitsuyoshi Abe, Masataka Mikai, Shuhei Kubota, Yutaka Fuchinoue, Sayaka Terazono, Kosuke Kondo, Naoyuki Harada, Nobuo Sugo

**Affiliations:** 1 Department of Neurosurgery (Omori), Toho University Graduate School of Medicine, Tokyo, JPN; 2 Department of Neurosurgery (Omori), Faculty of Medicine, Toho University, Tokyo, JPN

**Keywords:** regional cerebral blood flow, motor recovery, diffusion tensor image, corticospinal tract, brain tumor

## Abstract

Objective: To determine whether diffusion tensor image (DTI) parameters and regional cerebral blood flow (rCBF) serve to preoperatively predict postoperative motor outcomes in patients with brain tumors.

Methods: We included 81 patients with brain tumors who underwent surgical treatment. Motor function was assessed using the manual muscle test in the upper and lower limbs at admission and discharge. Fractional anisotropy (FA), mean diffusivity (MD), axial diffusivity (AD), radial diffusivity (RD), and their ratios (rFA, rMD, rAD, and rRD) were measured at the corona radiata, internal capsule, and cerebral peduncle of the corticospinal tract (CST). In addition, DTI and single photon emission computed tomography (SPECT) were synthesized to measure rCBF at the CST.

Result: Both DTI parameters and rCBF at the CST in the preoperative motor weakness group significantly differed from those of the preoperative normal function group. rFA at the cerebral peduncle and the internal capsule was considerably higher in those showing postoperative motor recovery than in those postoperative unchanged or with deteriorated motor function (P < 0.05). Moreover, there was significantly lower rMD and rRD at the internal capsule in the motor recovery group (P < 0.05, P < 0.01). Furthermore, rCBF was higher at all the cerebral peduncle, internal capsule, and corona radiate in the motor recovery group than in the unchanged and deteriorated motor function group (P < 0.05, P < 0.01, P < 0.01).

Conclusion: The analysis of DTI parameters and rCBF is useful in predicting postoperative motor outcomes in patients with brain tumors.

## Introduction

The prognosis of patients with brain tumors is mostly affected by the malignancy of the tumor itself and the surgical removal rate [1]. However, the level of postoperative neurological damage also plays an important role. Various neurological symptoms are associated with brain tumors, including aphasia, cranial nerve palsy, and sensory disorders, among which motor paralysis strongly limits patients' activities of daily life [2]. On the other hand, it is often difficult to clinically predict postoperative motor outcomes before surgery.

Recently, diffusion tensor imaging (DTI) is a technique that can produce three-dimensional images of nerve fibers using high-resolution MRI, enabling to visualize of nerve fibers in the corticospinal tract (CST) and other areas [3]. DTI is a type of diffusion-weighted image based on the diffusional motion of water molecules. In such images, water molecules diffuse spherically. On the other hand, if the direction of diffusion is restricted by axons or nerve fibers, water molecules diffuse elliptically. Thus, DTI evaluates diffusion anisotropy and can visualize nerve fibers. It has four parameters. Fractional anisotropy (FA) is the degree of anisotropy of diffusion, mean diffusivity (MD) is the magnitude of diffusion, axial diffusivity (AD) is the amount of diffusion in the long axis direction along nerve fibers, and radial diffusivity (RD) is the short-axis diffusivity perpendicular to the nerve fiber [3]. FA ranges from 0 in areas where water can diffuse freely, such as the cerebrospinal fluid, to 1 in areas where diffusion is restricted, such as the white matter, which has intermediate values in the gray matter. MD is not related to anisotropy and is small in areas where it cannot diffuse freely, such as white matter. While AD reflects axonal damage, RD indicates the integrity of the myelin sheath [4]. Accordingly, AD decreases and RD increases with nerve fiber damage [3].

Although numerous studies have reported the use of DTI in CST to predict motor prognosis in stroke patients [3,5-8], few studies have applied DTI and even fewer have measured the regional cerebral blood flow (rCBF) at CST on DTI to predict postoperative motor outcome in patients with brain tumors [9-11]. Neuroradiological imaging of MRI and CT can visualize morphological changes in the CST caused by edema and tumor invasion. Functional imaging modalities, such as positron emission tomography (PET) and single photon emission computed tomography (SPECT), are excellent for measuring rCBF. However, their low image resolution makes it difficult to identify the anatomy of the CST when its location changes due to the presence of brain tumors. Thus, we used DTI, which accurately assesses CST, and 123I-N-isopropyl-p-iodoamphetamine (123I-IMP) SPECT, which measures rCBF, to measure the rCBF at the CST on DTI. This study aimed to identify whether DTI parameters and rCBF can be used to preoperatively predict postoperative motor outcomes in patients with supratentorial brain tumors.

## Materials and methods

Patient selection

We recruited patients with supratentorial brain tumors who underwent surgical treatment at Toho University Medical Center Omori Hospital from January 2016 to May 2022. Among them, 81 patients (51 males and 30 females, age 62.7±12.8 years) who underwent DTI and SPECT imaging before surgery were included. The approach to tumor removal was chosen to minimize postoperative neurological damage. In no cases was the tumor localized to the bilateral CST on MRI or had received preoperative radiation therapy. This retrospective case-control study was approved by the Ethics Committee of Toho University Omori Hospital (Approval No. M22111). We gave the patients the opportunity to refuse to participate in the study.

DTI and MRI

Preoperative imaging was performed at 1.5T (GE MR Signa Excite HDxt Ver.15M4A, CT, USA) (Table 1). We used four DTI parameters (FA, MD, AD, and RD) as indicators for DTI quantification and set the location of the region of interest (ROI) in the image analysis of DTI parameters at the corona radiata, internal capsule, and cerebral peduncle of the CST. To prevent variability in ROI placement between patients, two board-certified neurosurgeons discussed and determined the area of the CST on the DTI and set the ROI. The FA, MD, AD, and RD values of CST on DTI were measured, and the affected/unaffected ratios (rFA, rMD, rAD, and rRD) were calculated. 

**Table 1:** Magnetic resonance image acquisition parameters. *Matsuura Author. TR: repetition time, TE: echo time, NEX: number of excitations, SR: slew rate.

Acquisition parameters	Measure
Acquisition technique	Echo planar imaging
TR/TE/NEX	13,000 ms/76.8 ms*
Field of view	240 mm
Section thickness	3 mm
Matrix	80 × 80
SR	120 mT/m/ms
Voxel size	0.33 × 0.33 × 3 mm
b	0 and 1000 s/mm^2 ^
Scanning time	7 min

SPECT imaging

**Figure 1:**
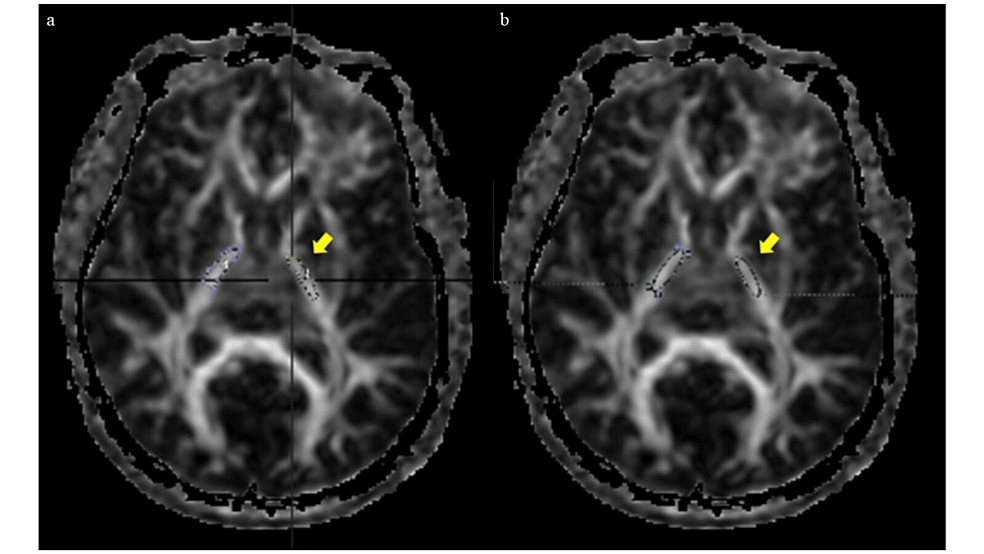
Example of how ROI is measured. (a) ROI of the internal capsule on DTI, (b) ROI of the internal capsule on synthesized DTI and SPECT. ROI: regions of interest, DTI: diffusion tensor image, SPECT: single photon emission computed tomography.

The SPECT system was a three-detector gamma camera (GCA-9300R™, Canon Medical Systems Corporation, Tochigi, Japan) (Table 2). DTI and SPECT were synthesized using a 3D image analysis system (Synapse Vincent Ver. 5.3, FUJIFILM, Tokyo, Japan) with normalized mutual information content [12]. In the synthesized images, rCBF was measured using the ROIs set by DTI (Figure 1). The rCBF of the cerebellar hemisphere ipsilateral to the lesion was measured as well.

**Table 2:** Collection condition of single photon emission computed tomography.

Acquisition parameters	Measure
Detector	Three-detector gamma camera
Collimator	Fan beam collimator (FANHR)
Matrix	128 × 128
Magnification	1.0
Pixel size	1.72 mm
Number of rotations	1
Sampling angle	4°
Acquisition mode	Step and shoot
Cycle	1
Acquisition time/view	50
Effective acquisition time	25 min
Reconstruction method	Butterworth 0.6–0.90 cycles/cm
Display window level (gray)	Upper: 100%–120%, lower: 5%–6%
Display window level (SP24)	Upper: 80%–110%, lower: 9%–11%
Display slice thickness	5.16 mm
Energy window	159 keV±10%

Clinical evaluation

The contralateral upper and lower extremities were evaluated using the manual muscle test (MMT) at admission and discharge (Table 3) [13]. The examiners measuring either DTI parameters or rCBF were blinded to MMT findings. Patients with a preoperative MMT of five were included in the preoperative normal function group, and patients with MMT ≤4 were classified into the preoperative motor weakness group. This group was further divided into a postoperative motor recovery group, a postoperative unchanged group, and a deteriorated motor function group. The postoperative motor recovery group was defined as cases in which MMT of either the upper or lower extremities improved by ≥1 level after surgery. The postoperative deteriorated motor function group was defined as those patients for whom MMT worsened by ≥1 level in either the upper or lower limb after surgery. The postoperative unchanged motor function group was defined as MMT, with no change in either upper or lower limbs before or after surgery.

**Table 3:** Manual muscle testing grades. This referenced paper is published under the Creative Commons (CC) license Attribution-NonCommercial-NoDerivatives 4.0 International (BY-NC-ND 4.0).

Grade	Criteria
5	Patient can hold position against maximum resistance and through the complete range of motion
4	Patient can hold position against strong to moderate resistance, with the full range of motion
3	Patient can tolerate no resistance but can perform the movement through the full range of motion
2	Patient has all or partial range of motion in the position with gravity eliminated
1	The muscle/muscles can be palpated while the patient is performing the action in the position with gravity eliminated
0	No contractile activity can be felt in the position with gravity eliminated

Image analysis

rFA, rMD, rAD, and rRD at the CST (the corona radiata, internal capsule, and cerebral peduncle) on the DTI were compared between the preoperative motor weakness group and the preoperative normal function group. rCBF at the CST on the DTI in both groups was also evaluated in the (affected/unaffected) and (affected/ipsilateral cerebellar hemispheres). rFA, rMD, rAD, rRD, and rCBF at the CST on the DTI were then compared with the postoperative motor recovery group and the postoperative unchanged and deteriorated motor function groups among the preoperative motor weakness group.

Statistical analysis

The statistical analysis software employed was IBM SPSS Statistics 28 (IBM Corp., NY, USA). The Mann-Whitney rank sum test was used to compare the two groups. Multiple comparisons were used to compare tumor location and pathologic classification between the preoperative motor weakness group and the normal function group and between the postoperative motor recovery group and the unchanged or deteriorated motor function group. We calculated Youden's index using receiver operating characteristic (ROCs) curves and area under the curves (AUCs) to determine cutoff values.

## Results

 Of the 81 patients, 32 were classified in the preoperative motor weakness group and 49 in the preoperative normal function group (Table 4). Patients were significantly older in the motor weakness group than in the normal function group (P < 0.01). In pathologic classification, there were more cases of meningioma in the normal function group than in the motor weakness group (P < 0.01). Comparing the DTI parameters in the normal function group with those in the motor weakness group (Table 5), rFA at the internal capsule and corona radiata were significantly lower in the motor weakness group (P < 0.05, P < 0.01), while rMD and rRD at the corona radiata were significantly higher (P < 0.01, P < 0.05), and rAD at the cerebral peduncle was significantly lower (P < 0.01). rCBF (affected/unaffected) at the internal capsule and corona radiata was significantly decreased in the preoperative motor weakness group than in the normal function group (both P < 0.01; Table 6). rCBF (affected/ipsilateral cerebellar hemisphere) at the cerebral peduncle, internal capsule, and corona radiata were significantly decreased in the motor weakness group (P < 0.05, P < 0.01, P < 0.01; Table 6). The cutoff value of rCBF (affected/ipsilateral cerebellar hemisphere) at the cerebral peduncle was 3.977 (sensitivity: 62.5%, specificity: 59.2%), at the internal capsule was 3.933 (sensitivity: 87.5%, specificity: 59.2%), and at the corona radiate was 3.209 (sensitivity: 78.1%, specificity: 71.4%).

**Table 4:** Summary of all cases. No.: number, side: tumor side, M: male, F: female, R: right, L: left, B: both, mean±SD (min-max): mean values±standard deviation (minimum-maximum). *The p-value was derived from the U test. †The p-value was derived from the Chi-square test.

	Paralysis (+)	Paralysis (-)	p-value
No. of patient	32	49	-
Age	Mean±SD (min-max) 70.4±10.1 (45–85)	Mean±SD (min-max) 57.7±12.1 (25–80)	<0.01*
Sex (M:F)	22:10	29:20	0.38†
Side (R:L:B)	17:15:0	20:22:7	0.07†
Site			0.49†
Frontal lobe	17	20	-
Temporal lobe	5	16	-
Parietal lobe	6	6	-
Occipital lobe	3	5	-
Suprasellar	1	2	-
Pathology			
Glioma	10	11	-
Meningioma	3	21	<0.01†
Metastatic brain tumor	14	13	-
Primary central nervous system lymphoma	5	1	-
Craniopharyngioma	0	1	-
Angioma	0	1	-
Ependymoma	0	1	-

**Table 5:** Comparison of preoperative motor weakness group and normal function group on diffusion tensor image. Paralysis (+): motor weakness group, paralysis (–): normal function group, FA: fractional anisotropy, MD: mean diffusivity, AD: axial diffusivity, RD: radial diffusivity, mean±SD: mean values±standard deviation, min: minimum, max: maximum.

	ROI	Paralysis (+)	Paralysis (-)	p-value	Cut-off	Sensitivity	Specificity	U-value
		Mean±SD (min-max)	Mean±SD (min-max)					
rFA	Cerebral peduncle	0.943±0.161 (0.571–1.213)	0.997±0.132 (0.758–1.413)	0.169	-	-	-	641.5
	Internal capsule	0.857±0.228 (0.323–1.452)	0.993±0.242 (0.741–2.462)	<0.05	0.893	0.563	0.816	518
	Corona radiata	0.718±0.351 (0.102–1.341)	0.962±0.236 (0.393–1.560)	<0.01	0.833	0.688	0.755	446.5
rMD	Cerebral peduncle	0.967±0.181 (0.446–1.475)	1.027±0.116 (0.813–1.351)	0.084	-	-	-	605
	Internal capsule	1.110±0.192 (0.905–1.639)	1.043±0.153 (0.608–1.468)	0.406	-	-	-	698
	Corona radiata	1.257±0.330 (0.909–2.247)	1.066±0.213 (0.644–1.787)	<0.01	1.034	0.719	0.674	496
rAD	Cerebral peduncle	0.930±0.128 (0.628–1.319)	1.017±0.112 (0.775–1.344)	<0.01	0.947	0.594	0.796	489
	Internal capsule	1.003±0.126 (0.833–1.452)	1.018±0.108 (0.720–1.368)	0.258	-	-	-	667
	Corona radiata	1.060±0.175 (0.797–1.457)	1.021±0.141 (0.778–1.481)	0.412	-	-	-	699
rRD	Cerebral peduncle	1.048±0.334 (0.280–2.059)	1.058±0.230 (0.545–1.722)	0.692	-	-	-	743
	Internal capsule	1.273±0.408 (0.807–2.279)	1.084±0.236 (0.485–1.735)	0.072	-	-	-	597.5
	Corona radiata	1.478±0.661 (0.780–3.650)	1.134±0.370 (0.533–2.622)	<0.05	1.14	0.563	0.755	534

**Table 6:** Comparison of preoperative motor weakness group and normal function group on single photon emission computed tomography. Paralysis (+): preoperative motor weakness group, paralysis (–): preoperative normal function group, rCBF (A/S): regional cerebral blood flow (affected/unaffected), rCBF (A/IC): regional cerebral blood flow (affected/ipsilateral cerebellar hemisphere), mean±SD: mean values±standard deviation, min: minimum, max: maximum.

	ROI	Paralysis(+)	Paralysis (-)	p-value	Cut-off	Sensitivity	Specificity	U-value
		Mean±SD (min-max)	Mean±SD (min-max)					
rCBF (A/S)	Cerebral peduncle	1.007±0.313 (0.294–1.724)	1.110±0.518 (0.184–2.851)	0.824	-	-	-	761
										Internal capsule	0.740±0.371 (0.098–1.053)	1.080±0.501 (0.414–3.259)	<0.01	0.816	0.656	0.755	439
	Corona radiata	0.756±0.558 (0.067–2.119)	1.147±0.707 (0.100–3.370)	<0.01	0.792	0.625	0.694	508									
									rCBF (A/IC)	Cerebral peduncle	4.101±2.448 (0.610–11.960)	5.661±2.963 (0.481–12.241)	<0.05	3.977	0.625	0.592	531
	Internal capsule	2.881±2.031 (0.161–11.360)	4.756±2.637 (1.452–11.727)	<0.01	3.933	0.875	0.592	408									
										Corona radiata	2.163±1.366 (0.278–5.333)	4.422±2.278 (0.561–13.036)	<0.01	3.209	0.781	0.714	285.5

Of the 32 patients in the preoperative motor weakness group, 13 had improved paralysis and 19 patients had unchanged or deteriorated paralysis (Table 7). Comparing the DTI parameters in the postoperative motor recovery group with those in the unchanged or deteriorated motor function group (Table 8), rFA at the cerebral peduncle and the internal capsule was significantly higher (P < 0.05, P < 0.05) and rMD and rRD at the internal capsule were significantly lower (P < 0.05, P <0.01) in the motor recovery group. The cutoff value of rFA at the cerebral peduncle was 0.914 (sensitivity: 63.2%, specificity: 76.9%), and that of rFA at the internal capsule was 0.926 (sensitivity: 84.2%, specificity: 61.5%) (Table 8). rCBF (affected/unaffected) was significantly increased (P < 0.01, P < 0.05) at the internal capsule and the corona radiata in the motor recovery group compared to the unchanged and deteriorated motor function groups (Table 9). With respect to the rCBF (affected/ipsilateral cerebellar hemispheres), the motor recovery group had a significantly increased rCBF compared to the unchanged or deteriorated motor function group at all the cerebral peduncle, internal capsule, and corona radiata (P < 0.05, P < 0.01, P < 0.01). The cutoff value of rCBF (affected/ipsilateral cerebellar hemisphere) at the cerebral peduncle was 3.977 (sensitivity: 84.2%, specificity: 69.2%), at the internal capsule was 3.433 (sensitivity: 94.7%, specificity: 61.5%), and at the corona radiate was 2.511 (sensitivity: 89.5%, specificity: 76.9%). These values were lower than comparative values in the preoperative motor weakness group and the normal function group.

**Table 7:** Summary of all cases of paralysis. No.: number, side: tumor side, M: male, F: female, R: right, L: left, improvement: group with postoperative improvement in motor weakness, unchanged: group with no postoperative change in motor weakness, deteriorated: group with postoperative deterioration of motor weakness, mean±SD (min-max): mean values±standard deviation (minimum-maximum).

	Improvement	Unchanged+deteriorated
No. of patient	13	19
Age	Mean±SD (min-max) 71.1±11.0 (51–85)	Mean±SD (min-max) 69.9±9.7 (45–85)
Sex (M:F)	8:5	14:5
Side (R:L)	6:7	11:8
Site		
Frontal lobe	7	10
Temporal lobe	0	5
Parietal lobe	5	1
Occipital lobe	1	2
Suprasellar	0	1
Pathology		
Glioma	3	7
Meningioma	2	1
Metastatic brain tumor	7	7
Primary central nervous system lymphoma	1	4
Craniopharyngioma	0	0
Angioma	0	0
Ependymoma	0	0

**Table 8:** Comparison of postoperative improvement group and unchanged or deterioration on diffusion tensor image. FA: fractional anisotropy, MD: mean diffusivity, AD: axial diffusivity, RD: radial diffusivity, improvement: group with postoperative improvement in motor weakness, unchanged: group with no postoperative change in motor weakness, deteriorated: group with postoperative deterioration of motor weakness, mean±SD: mean values±standard deviation of mean, min: minimum, max: maximum.

	ROI	Improvement	Unchanged+deteriorated	p-value	Cut-off	Sensitivity	Specificity	U-value
		Mean±SD (min-max)	Mean±SD (min-max)					
rFA	Cerebral peduncle	1.015 ±0.130 (0.806–1.196)	0.894±0.164 (0.571-1.213)	<0.05	0.914	0.632	0.769	69
	Internal capsule	0.963±0.211 (0.545–1.452)	0.785±0.216 (0.323–1.171)	<0.05	0.926	0.842	0.615	68
	Corona radiata	0.822±0.324 (0.317–1.341)	0.647±0.360 (0.102–1.278)	0.15	-	-	-	86
rMD	Cerebral peduncle	0.901±0.173 (0.446–1.121)	1.012±0.177 (0.763–1.475)	0.205	-	-	-	90.5
	Internal capsule	1.015±0.101 (0.905–1.329)	1.175±0.213 (0.907–1.639)	<0.05	1.042	0.737	0.923	64
	Corona radiata	1.158±0.256 (0.909–1.630)	1.325±0.363 (0.919–2.247)	0.111	-	-	-	82
rAD	Cerebral peduncle	0.912±0.124 (0.628–1.065)	0.943±0.133 (0.763–1.319)	0.774	-	-	-	116
	Internal capsule	0.978±0.083 (0.846–1.186)	1.020±0.149 (0.833–1.452)	0.604	-	-	-	110
	Corona radiata	1.027±0.142 (0.876–1.325)	1.083±0.195 (0.797–1.457)	0.388	-	-	-	101
rRD	Cerebral peduncle	0.910±0.274 (0.280–1.326)	1.142±0.345 (0.649–2.059)	0.111	-	-	-	82
	Internal capsule	1.086±0.280 (0.807–1.953)	1.402±0.438 (0.837–2.279)	<0.01	1.143	0.684	0.846	55
	Corona radiata	1.325±0.461 (0.780–2.255)	1.582±0.764 (0.795–3.650)	0.454	-	-	-	104

**Table 9:** Comparison of postoperative improvement group and unchanged or deterioration on single photon emission computed tomography. rCBF (A/S): regional cerebral blood flow (affected/unaffected), rCBF (A/IC): regional cerebral blood flow (affected/ipsilateral cerebellar hemisphere), mean±SD: mean values±standard deviation of mean, min: minimum, max: maximum.

	ROI	Improvement	Unchanged+deteriorated	p-value	Cut-off	Sensitivity	Specificity	U-value
		Mean±SD (min-max)	Mean±SD (min-max)					
rCBF (A/S)	Cerebral peduncle	1.077±0.233 (0.619–1.457)	0.960±0.356 (0.294–1.724)	0.242	-	-	-	93
										Internal capsule	0.933±0.275 (0.490–1.503)	0.608±0.376 (0.098–1.493)	<0.01	0.774	0.737	0.769	54
	Corona radiata	1.060±0.626 (0.265–2.119)	0.548±0.405 (0.067–1.253)	<0.05	0.533	0.632	0.769	67									
									rCBF (A/IC)	Cerebral peduncle	5.358±2.795 (2.000–11.960)	3.241±1.786 (0.610–8.596)	<0.05	3.977	0.842	0.692	58
	Internal capsule	4.062±2.577 (1.563–11.360)	2.074±0.992 (0.161–3.740)	<0.01	3.433	0.947	0.615	46									
										Corona radiata	3.201±1.237 (1.254–5.333)	1.452±0.939 (0.278–3.567)	<0.01	2.511	0.895	0.769	33

## Discussion

DTI parameters are an important marker of CST damage due to brain tumors and are reported to be worse in patients with motor weakness [10,11,14]. In our study, the DTI parameters at the CST of patients with motor weakness significantly differed from those of patients with normal function. Structural changes at the CST due to brain tumors are displacement, edematous, infiltrated, and disrupted [15]. Witwer et al. defined them as follows: displaced, normal anisotropy relative to the corresponding tract in the contralateral hemisphere but situated in an abnormal location or with an abnormal orientation on color-coded orientation maps; edematous, normal anisotropy and orientation but high signal intensity on T2-weighted MR images; infiltrated, reduced anisotropy but identifiable on orientation maps; and disrupted, markedly reduced anisotropy such that the tract could not be identified on orientation maps. Patients with motor weakness have significantly more displaced CST and a shorter distance between the CST and the tumor [11]. Such structural changes at the CST on DTI reflect pathological changes [16]. Bakhshi et al. reported that patients with intracranial brain tumors who presented with infiltration as structural changes had a worse prognosis and tended to have more malignant gliomas [9]. In meningiomas, the structure of nerve fibers is maintained even when the CST is compressed [17]. In our study, there were significantly more meningioma cases in the normal function group than in the motor weakness group. It was suggested that the despite displacement of the CST and changes in DTI parameters, nerve fiber structure was maintained and patients did not present with motor weakness. Thus, even though the DTI parameters at the CST worsened due to compression from the brain tumor, the normal white matter function resulted in patients having no motor weakness [18,19].

In our study, we found that rCBF at the CST was significantly decreased in the motor weakness group compared to the normal function group. This suggests that motor weakness caused by brain tumors is strongly influenced by a decrease in rCBF as well as changes in DTI parameters at the CST. Therefore, evaluating rCBF and DTI parameters would be useful for further clarifying the mechanism underlying patients’ motor weakness.

Moreover, patients with motor weakness were significantly older than those with normal function in the present study. CBF decreases with age due to deteriorated glucose metabolism and decreased oxygen supply [20]. Therefore, it is possible that older patients were more affected by the reduced CBF caused by the brain tumor and more likely to develop paralysis.

Here, we compared the postoperative motor recovery group with the unchanged or deteriorated motor function group and found a significant difference in DTI parameters at the preoperative CST between groups. Although no previous reports used DTI parameters at the CST to predict postoperative motor outcomes in patients with brain tumors, Laundre et al. reported that pre-and postoperative structural changes in DTI at the CST correlated strongly with postoperative motor outcomes [10]. They also described normalization of the CST position and anisotropy postoperatively, resulting in improvement of motor weakness [10]. In our study, since the images were not re-evaluated by DTI after surgery, imaging changes over time are unknown, but we suspect that motor weakness improved by a similar mechanism as that proposed by Laundre et al.

Puig et al. reported that rFA at the CST measured at 30 days after stroke onset correlated with motor outcome in stroke patients, with a cutoff value of 0.925 (sensitivity: 95.2%, specificity: 94.9%) [5]. In our research, the cutoff value of rFA at the cerebral peduncle was 0.914 (sensitivity: 63.2%, specificity: 76.9%), and that of rFA at the internal capsule was 0.926 (sensitivity: 84.2%, specificity: 61.5%), which is similar to their reported result. Thus, the rFA may be a useful prognostic predictor of motor outcomes in patients with brain tumors.

In addition, the higher the preoperative rCBF value was maintained at the CST, the more likely it was that paralysis would be improved postoperatively. The cortical hypoperfusion associated with brain tumors is caused by ischemia and metabolic depression [21]. The postoperative CBF recovery may be due to a decrease in intracranial pressure caused by tumor removal and a reduction in peritumoral edema [22]. This suggests that postoperative paralysis was improved by the normalization of CST displacement after tumor removal and the improved rCBF indicated by the recovery of the DTI parameters. Additionally, a high CBF level was maintained in the motor recovery group, which possibly maintained the integrity of their neuron fibers. Thus, the evaluation of the preoperative rCBF, as well as that of DTI parameters, may be a predictive indicator for motor outcomes in such patients. 

Limitations

One limitation is that we did not evaluate each pathological classification or examine the site of origin. Therefore, more detailed studies on tumor grade and location in relation to CST are needed. PET-MRI can easily provide both functional and morphological images at the same time, but it is an expensive technology, and there are a limited number of facilities where it is available. Thus, the image combination in this study is an acceptable method that can be performed in a relatively short time with high accuracy using 3D image analysis software.

## Conclusions

We evaluated DTI parameters and rCBF at the CST in patients with supratentorial brain tumors and investigated their relationship to motor weakness. The higher preoperative rFA is maintained at the cerebral peduncle and the internal capsule, and the lower preoperative rMD and rRD are maintained at the internal capsule, the more likely it is that the paralysis will improve after surgery. Moreover, the higher the preoperative rCBF value is maintained at the CST, the more likely paralysis will improve after surgery. Therefore, analysis of DTI parameters and rCBF is useful in predicting postoperative motor outcomes in patients with brain tumors. We recommend that neurosurgeons and radiologists use not only DTI but also SPECT for preoperative evaluation of the CST.
